# PSMA PET as a Tool for Active Surveillance of Prostate Cancer—Where Are We at?

**DOI:** 10.3390/jcm14103580

**Published:** 2025-05-20

**Authors:** Jonathon Carll, Jacinta Bonaddio, Nathan Lawrentschuk

**Affiliations:** 1 Department of Surgery, University of Melbourne, Melbourne, VIC 3052, Australia; 2 Department of Urology, The Royal Melbourne Hospital, Melbourne, VIC 3052, Australia; 3 EJ Whitten Prostate Cancer Research Centre, Epworth Healthcare, Melbourne, VIC 3005, Australia; 4 Department of Surgery, Peter MacCallum Cancer Centre, Melbourne, VIC 3052, Australia

**Keywords:** PSMA PET, prostate cancer, active surveillance, risk stratification

## Abstract

Active surveillance remains the preferred treatment for men with low-risk prostate cancer and select men with favourable intermediate-risk prostate cancer. It involves the close observation of clinicopathological parameters to assess for disease progression, aiming to delay or avoid definitive treatment and related toxicities for as long as possible, without compromising oncological outcomes. A recent advancement in prostate cancer staging is the PSMA PET scan, which uses a tracer that strongly binds a highly expressed cellular biomarker for prostate cancer. Recent articles have also demonstrated that PSMA PET may be a useful tool for risk-stratifying prostate cancer, with the SUVMax of the scan correlated with higher-grade prostate cancer. This has ignited interest in the potential use of PSMA PET to identify men with higher-risk prostate cancer who may be unsuitable for active surveillance, particularly those who were incorrectly classified as lower risk upon initial diagnosis. This review article aims to assess the current state of the literature and clinical guidelines regarding the use of PSMA PET as a tool to risk-stratify prostate cancer, and whether it can be incorporated into active surveillance protocols to identify men who were incorrectly risk-stratified at time of initial diagnosis.

## 1. Introduction

Prostate cancer is the second most commonly diagnosed cancer worldwide [1], with its incidence increasing with age. However, a significant proportion of patients with prostate cancer will die from other causes unrelated to the disease [2]. Large autopsy-based and epidemiological studies have confirmed that many men will die with prostate cancer, rather than from it [3,4]. In the era of widespread PSA-based detection of early prostate cancer, significant concerns have been raised regarding the over-diagnosis and over-treatment of lower-grade prostate cancers that may not lead to patient death within their natural lifetime [5]. Given the significant toxicities from conventional surgical and radiotherapy treatments, modern guidelines suggest a risk-adapted approach to managing prostate cancer, with a significant proportion of patients with low-risk cancers able to defer upfront treatment in favour of a period of active surveillance [6,7].

Active surveillance of prostate cancer is a curative approach to low-risk and select intermediate-risk cases [8], aiming to delay significant interventions, such as prostatectomy or radiotherapy, without compromising oncological outcomes. It involves regular PSA monitoring, repeat digital rectal examinations (DREs), and repeat prostate biopsies to assess for progression to higher-risk prostate cancer that may require definitive treatment [9]. Repeat biopsies are an essential part of all active surveillance programs that do not include initial MRI-targeted and systemic biopsies [8,9], as core needle biopsies used for the prostate cancer diagnosis can under-sample or miss areas of higher-grade cancer [10,11]. This can result in the cancer being incorrectly classified as lower-risk disease [9]. Multiparametric MRI (mpMRI) is a major advancement in the detection and diagnosis of early prostate cancer. It enables more accurate T staging [12] and more precise targeting of suspicious prostate lesions [13], thereby increasing biopsy sensitivity [14]. This enhances the detection of clinically significant prostate cancer while reducing the detection of lower-grade disease [13]. As a result, all major guidelines now recommend incorporating mpMRI into active surveillance if it was not used to guide the initial prostate biopsy [6,7,8].

Active surveillance is suitable for patients with low-risk prostate cancer and select patients with favourable intermediate-risk prostate cancer based on risk stratification [8], which considers various clinicopathological features, including pathological grade group, PSA level, and clinical T stage. Pathologically, risk stratification is based on the grade groups defined by the International Society of Urological Pathology (ISUP) [15]. These were adapted from the older Gleason scoring system, where tumours glands were graded from one to five based on the degree of pathological differentiation, with a final score derived from the two most predominant patterns [15,16]. Major urological guidelines define low-risk and favourable intermediate risk prostate cancer to include ISUP Grade Groups 1-2 with low PSAs, T stage less than 3 and low-volume of disease. [6,7,8]. As such, the accurate pathological grading of a tumour is essential for selecting men suitable for active surveillance. However, a certain proportion of men may be misidentified with a lower pathological grade group upon initial biopsy due to inadequate or inaccurate sampling of the primary tumour [8,10,11]. As such, men undergoing active surveillance should undergo a confirmatory biopsy to ensure that the initially diagnosed tumour is appropriate for this approach [8].

**Figure 1:**
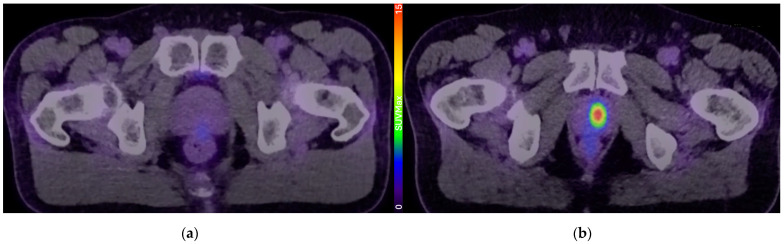
Example PSMA PET/CT scans demonstrating intra-prostatic uptake of radiolabelled PSMA tracer. (**a**) PSMA scan demonstrating low intraprostatic uptake—PRIMARY score of 1; (**b**) PSMA scan demonstrating a focal area of high tracer uptake—PRIMARY score of 5.

A more recent advancement is the PSMA PET scan, which uses a radiolabelled tracer that binds to PSMA, a type II transmembrane glycoprotein that is heavily over-expressed in most prostate cancers [17]. It has revolutionised the staging of high-risk prostate cancer and is far more sensitive and specific than conventional imaging modalities in detecting distant lymph nodes and metastasis [18]. More recently, there has been significant interest in the use of PSMA PET for detection and risk stratification in localised prostate cancer. A growing body of evidence has demonstrated that the maximum standardised uptake value (SUVMax) of the PSMA PET scan is associated with higher proportion of pattern 4 disease and unfavourable pathology [19,20]. The PRIMARY study demonstrated that the addition of PSMA PET can improve the sensitivity and negative predictive value of an MRI [21], and that a 5-point PRIMARY score can provide high diagnostic accuracy for clinically significant (Grade Group 2 and above) prostate cancer [22]. As such, there has been considerable interest in whether PSMA PET can be incorporated into active surveillance protocols to improve risk stratification or exclude patients based on high-risk imaging findings (Figure 1). 

The PSMA PET scan was primarily developed as a staging scan, enabling the highly accurate detection of local and distant metastatic disease. This would provide clinicians with crucial information prior to deciding on definitive treatments, such as radiation therapy or radical prostatectomy, which can have significant clinical impact on how a patient is managed. Given the high specificity of PSMA PET for detecting nodal metastasis [23], it has become an essential tool in the work-up of newly diagnosed prostate cancer patients prior to definitive management [24]. This is particularly relevant for patients undergoing prostatectomy. One study found that if a negative PSMA PET scan was used as the basis for not performing a pelvic lymph node dissection, 80% of men would avoid an unnecessary procedure [25]. This has sparked considerable debate, given the significant morbidity associated with the procedure [26], as well as the findings of a large systematic review that did not demonstrate any oncological benefits to lymph node dissection [27]. While pelvic lymph node dissection remains the gold standard for local staging, PSMA PET has become increasingly useful in determining which patients do not require the procedure during radical prostatectomy [23].

PSMA PET has also demonstrated significant utility in managing patients with biochemical recurrence of prostate cancer after definitive treatment. In patients previously treated with radical prostatectomy, a PSMA PET scan can detect active disease in 42% of patients with a PSA < 0.2, with disease detection rates rising to >95% in patients with a PSA > 2.0 [28]. This can help guide clinical decision-making, with a systematic review finding that it changes management in as many as 56% of cases [29]. Given the potential curative treatment with salvage radiotherapy, a PSMA PET scan is a useful tool in detecting metastasis distant from the prostate bed and can help guide metastasis-directed radiotherapy in suitable patients. PSMA PET also plays an important role in the emerging area of radioligand therapy. Lu-177 PSMA is a radioligand therapy that delivers radioactive Lu-177 conjugated with an antibody specific for PSMA, allowing for the targeted delivery of the radioisotope to cells that heavily express PSMA [30]. As it has recently shown clinical utility in patients with metastatic prostate cancer [31,32,33], Lu-177 PSMA has become the first radioligand therapy approved for treatment of the disease. It is therefore essential for a baseline PSMA PET scan to be conducted prior to initiating treatment, as cancers without PSMA expression may not be adequately targeted by Lu-177 PMSA therapy [34]. PSMA PET has become a routine part of clinical practice in staging newly diagnosed prostate cancers and managing advanced stages of disease. It also has an emerging role in early prostate cancer, which we will explore in this review.

## 2. Materials and Methods

In this narrative review, the current evidence supporting the use of PSMA PET in guiding active surveillance protocols will be reviewed and discussed. Key articles examining the ability of PSMA PET to risk-stratify prostate cancer, as well as the publications examining its potential incorporation into active surveillance algorithms, will be reviewed and discussed.

To identify studies for inclusion in this review, we used the key words “PSMA PET” AND “Active Surveillance” AND “Prostate Cancer” to search PubMed. A total of 42 articles were found and screened for relevance to the review. Additional reference screening was undertaken to identify key papers and ongoing trials. Key international guidelines on uro-oncology were also reviewed to identify any current recommendations or consensus regarding the use of PSMA PET in active surveillance. Relevant English language papers were synthesized to develop a cohesive narrative regarding the current applicability and potential future use of PSMA PET in the active surveillance of prostate cancer.

## 3. Risk Stratification Using PSMA PET

There is a growing body of evidence supporting the potential utility of PSMA PET in risk-stratifying patients with prostate cancer. A large retrospective cohort of 1123 patients who underwent a PSMA PET scan followed by radical prostatectomy demonstrated a strong correlation between SUVMax on the PSMA PET and the likelihood of finding Grade Group 3 prostate cancer [20]. As ISUP Grade Group 3 prostate cancer is more aggressive and unsuitable for active surveillance, the authors suggested that patients with an intraprostatic lesion and an SUVMax >11 should not undergo active surveillance without a targeted biopsy of the lesion, as it is very likely to be a higher-grade cancer. Conversely, an SUVMax of <5 is strongly associated with a low likelihood of pathological upgrading to a grade higher than Grade Group 2 at the time of radical prostatectomy. The retrospective analysis also confirmed the findings of previous studies, which demonstrated that PSMA PET has an additive value and increases the negative predictive value and sensitivity for clinically significant prostate cancer when combined with mpMRI.

This is the largest patient cohort from a growing number of papers examining the use of PSMA PET in risk-stratifying men with prostate cancer. A systematic review identified ten such papers, mostly retrospective, demonstrating a number of key findings [35]. PSMA PET was shown to be highly accurate and effective in staging prostate cancer and is a useful tool in guiding treatment strategies [36], changing the management approach in 28.7% of patients in one study [37]. Another study by Xue et al. also demonstrated that an increased SUVMax on a PSMA PET is associated with a higher proportion of Gleason 4 disease and adverse pathology at the time of radical prostatectomy [19]. This is a very important finding, as the ISUP grade group for intermediate-risk prostate cancers is determined by the percentage of Gleason pattern 4 disease. ISUP Grade Group 2 prostate cancers, which may be suitable for active surveillance, by definition, have predominately Gleason pattern 3 disease, with only a small proportion of pattern 4 disease. ISUP Grade Group 2 prostate cancers with higher percentages of pattern 4 disease may also be unsuitable for active surveillance. This finding suggests that PSMA PET could play an important role in risk-stratifying men with newly diagnosed prostate cancer and identifying patients who are unsuitable for an active surveillance approach. Overall, there is a growing body of evidence demonstrating that PSMA can provide effective staging for prostate cancer, guide targeted biopsies of highly active lesions, and detect MRI-occult lesions, and that a high SUVMax is associated with adverse pathological features, which would make a patient unsuitable for active surveillance.

## 4. Current Studies Examining PSMA PET in Active Surveillance

To date, there have been three published studies specifically examining the use of PSMA PET scans in active surveillance protocols [38,39,40]. Two were prospective [38,39], and one was retrospective [40], enrolling 211 patients in total. Pepe et al. [38] enrolled 40 men with low-risk prostate cancer who had already been on active surveillance for 48–60 months to undergo PSMA PET and mpMRI prior to a scheduled repeat biopsy. In this cohort, PSMA PET did not improve the detection of clinically significant prostate cancer but was superior to mpMRI in identifying patients who could be safely excluded from a repeat biopsy, demonstrating a lower false positive rate. This is an important finding; however, it should be interpreted within the appropriate context. PSMA PET and mpMRI have different advantages and disadvantages when used for long-term surveillance. mpMRI is beneficial in that it avoids radiation exposure, which is crucial given the multiple scans that may be needed over a period of surveillance. However, MRI has a more significant list of contraindications, which can limit its implementation in patients with implants, foreign bodies, claustrophobia, or renal function that is too impaired for the administration of gadolinium-based contrast. Ultimately, the evidence is still lacking regarding which modality is better for implementation into surveillance protocols based on cost, diagnostic accuracy, and patient adherence.

The PASPoRT trial by Heetman et al. [39] enrolled 141 patients with newly diagnosed low-risk or favourable intermediate-risk prostate cancer to undergo PSMA PET while on active surveillance. The scan detected 45 (32% of patients) additional PSMA-avid lesions for targeted biopsy, resulting in 14 (9%) of the patients having their pathological grade group the number needed to scan to detect one patient with higher grade group disease was 11. While a promising prospective study, it had several limitations. The MRI was not centrally read, leading to variability within the study and the potential for high-grade lesions to be missed on the initial scan. It included all men going for active surveillance, not just men with high-risk features. It also used an SUVMax of four as a cut off for a targeted biopsy, rather than a formalized reporting system, such as the PRIMARY score. While promising, this study is far from definitive, and PSMA PET may be more useful if used in a better-selected group of patients undergoing active surveillance.

A retrospective study performed by Jain et al. [40] presented a single-centre, single-surgeon model. They examined 30 patients with newly diagnosed low-risk or favourable intermediate-risk prostate cancer who had undergone PSMA PET. Notably, 11 of the 30 men were on active surveillance, with only 1 converting to radical treatment. Overall, 15 (50%) patients had a concerning lesion on PSMA PET, with 9 (60%) of these patients having concerning histology on the final radical prostatectomy. This small-scale study demonstrated that there is a growing role for PSMA in risk-stratifying and identifying patients at risk of having adverse pathology, despite its limited statistical power (Table 1).

## 5. Ongoing Prospective Trials

Whiste there are some promising results in the literature, high-powered, large-volume studies assessing the utility of PSMA PET during active surveillance are lacking. This remains an ongoing area of research, likely to develop in the coming years. Our review of the literature identified two large prospective trials evaluating the role of PSMA PET in the active surveillance of prostate cancer, both of which are currently recruiting. The PIAS Study [41] is seeking to enrol 225 newly diagnosed prostate cancer patients, including patients with ISUP Grade 1 prostate cancer with high-risk features, as well as those with favourable intermediate-risk prostate cancer. The patients will undergo PSMA PET and mpMRI within 3 months of a confirmatory saturation biopsy, with the primary outcome being the ability of PSMA PET to detect or exclude clinically significant prostate cancer at the time of a repeat biopsy. The study also aims to repeat mpMRI and PSMA PET scans for patients who remain on active surveillance at the 3–4-year mark. This will be a highly powered, prospective, cross-sectional, partially blinded multicentre trial, which should provide significant data on any potential benefits of PSMA PET for men undergoing active surveillance.

Similarly, the CONFIRM trial is another prospective trial involving PSMA PET in active surveillance that is currently actively recruiting patients [42,43]. It aims to recruit 223 men with recently diagnosed low-risk prostate cancer exhibiting high-risk features, such as ISUP Grade 1 prostate cancers with high volume, concerning MRI features, high PSA or high PSA density, or ISUP Grade 2 cancers with low pattern 4 disease. A PSMA PET is performed alongside a repeat MRI scan, followed by a confirmatory biopsy at 6 months. Additional targeted biopsies will be taken from any PSMA-avid lesions, along with MRI-targeted and systemic biopsies. The primary endpoints will include the proportion of men deemed unsuitable for ongoing AS based on both pathological upgrading and multidisciplinary team recommendations resulting from the PSMA PET/CT scans and PSMA PET-targeted confirmatory biopsy, as well as the NPV of a pelvic PSMA PET scan. Preliminary data from the first 60 patients enrolled in the trial have been promising, with 45.5% of patients showing MRI-occult lesions on the PSMA PET, and 40% of the MRI-occult lesions harbouring higher-grade disease than the initial biopsy [43]. The final results of this study should provide clear evidence as to whether PSMA PET adds value in this cohort of men undergoing active surveillance. It should help determine if PSMA PET can detect higher-grade lesions missed by the initial MRI-guided biopsy and whether it is a useful tool for identifying patients unsuitable for active surveillance.

## 6. Current Guidelines

While PSMA PET is a promising technology that may someday be more routinely implemented into active surveillance protocols, the evidence supporting its use has yet to be definitively established. As such, there are no strong recommendations from major guidelines regarding the use of PSMA PET in active surveillance. The AUA guidelines on localised prostate cancer do not discuss the use of PSMA PET in relation to active surveillance at all [7]. The latest version of the EAU guidelines makes only a brief reference to PSMA PET in active surveillance, stating the following: “A few studies indicate that PSMA-PET-CT or PSMA-PET-MRI may have additional value to the above-mentioned clinico-pathological variables for risk stratification before AS. However, so far, the studies are too small and the follow-up too short to draw any hard conclusions for this modality to be recommended outside of clinical trials” [6].

The National Comprehensive Cancer Network^®^ (NCCN^®^) Guidelines for Prostate Cancer also do not directly discuss any role of PSMA PET in active surveillance [44]. The only reference to PSMA PET in active surveillance is actively discouraging the use of scans for metastatic staging, stating the following: “A metastatic staging evaluation (PSMA PET, bone scan, CT scan, or whole body MRI) should not be performed [44]”.

These guidelines reflect the immaturity of the current evidence for PSMA PET in active surveillance. There are promising early data, particularly regarding PSMA’s ability to accurately risk-stratify patients with localised prostate cancer and predict the volume of pattern 4 disease. However, this evidence is currently insufficient for major professional associations to make definitive guidelines supporting its routine use in active surveillance. Until the utility of PSMA PET in active surveillance is established within a clinical setting, this is likely to remain the case. The PRIAS and CONFIRM trials are the two most promising ongoing studies, which may help clarify the role of PSMA PET in active surveillance. Until the results from these studies are published, it is likely that the guidelines will remain unchanged.

## 7. Conclusions

Active surveillance remains the preferred management approach to low-risk and favourable intermediate-risk prostate cancers. Key to the effectiveness of active surveillance protocols is the correct selection of patients and the use of a confirmatory biopsy to exclude missed higher-grade cancer that would be unsuitable for surveillance. This is an evolving field, with mpMRI being the latest innovation incorporated into active surveillance protocols. The PSMA PET scan has also shown promise in risk-stratifying men with newly diagnosed prostate cancer, particularly in identifying cancers with a higher percentage of Gleason pattern 4 disease. This has potential implications for active surveillance, as it could enhance the detection of men who may not be suitable for this approach, as well as enable more targeted confirmatory biopsies to ensure that men on active surveillance do not harbour occult higher-grade disease. However, to date, there has only been one prospective trial and two smaller retrospective studies assessing the use of PSMA PET in active surveillance. Therefore, it remains a promising concept that has yet to accumulate enough high-grade evidence to support its routine clinical use. Fortunately, there are currently at least two ongoing prospective trials assessing the utility of PSMA PET in active surveillance protocols, both of which are focused on enrolling men with higher-risk features for active surveillance. These trials, along with other further research, will hopefully determine whether incorporating PSMA PET into active surveillance protocols provides significant clinical utility.

## Figures and Tables

**Table 1:** Papers that have examined PSMA PET in active surveillance.

Paper	Study Design	Patients (N)	Inclusion Criteria	PET Timing	Key Findings
Pepe et al. [38]	Prospective	40	Very-low-risk prostate cancer patients on active surveillance	48–60 months after initial diagnosis	PSMA PET is superior to MRI at detecting patients that can safely be excluded from a repeat biopsy.
Heetman et al. [39]	Prospective	141	Newly diagnosed low-risk or favourable intermediate-risk disease suitable for active surveillance	6 months after initial diagnosis	Up to 9% of patients had pathological upgrading detected on PSMA PET-targeted biopsy.
Jain et al. [40]	Retrospective	30	Newly diagnosed low-risk or favourable intermediate-risk disease suitable for active surveillance		50% of men suitable for active surveillance had concerning findings on PSMA PET. PSMA can help inform management of men considering active surveillance.

## Abbreviations

The following abbreviations are used in this manuscript:

PSMA	Prostate-Specific Membrane Antigen
PET	Positron Emission Tomography
mpMRI	Multi-parametric MRI
MRI	Magnetic Resonance Imaging
ISUP	International Society of Urological Pathology
SUVMax	Maximum Standardized Uptake Value
AUA	American Urological Association
EAU	European Association of Urology
NCCN	National Comprehensive Cancer Network
